# Coloration in a Praying Mantis: Color Change, Sexual Color Dimorphism, and Possible Camouflage Strategies

**DOI:** 10.1002/ece3.70398

**Published:** 2025-01-06

**Authors:** Leah Y. Rosenheim, Jay A. Rosenheim, Michael R. Maxwell

**Affiliations:** ^1^ Department of Biological Sciences Binghamton University Binghamton New York USA; ^2^ Department of Entomology and Nematology University of California Davis Davis California USA; ^3^ Department of Mathematics and Natural Sciences National University San Diego California USA

**Keywords:** background choice, background matching, crypsis, generalist coloration, mobility, sexual dichromatism

## Abstract

Background matching, an important form of camouflage, can be challenging for animals that range across heterogeneously colored habitats. To remain cryptic in such habitats, animals may employ color change, background choice, or generalist coloration, and the efficacy of these strategies may be influenced by an animal's mobility. We examined camouflage strategies in the praying mantis *Stagmomantis limbata*. We reared mantids in green or brown containers to test whether mantids change color over development to match their background. Additionally, we tested whether adult mantids (i) employ behavioral background choice, (ii) exhibit sexual color dimorphism, and (iii) differ in mobility in the field. Mantids changed color during development in response to their background, but the effect was small and variable. Adult mantids did not show background choice. In the field, adult males moved greater distances than females. Adults exhibited sexual color dimorphism: Males were heterogeneous in coloration (green body with brown pronotum), while females were more homogeneous in color, ranging continuously from green to brown. We suggest a hypothesis that differences in mobility between the sexes have led to the sexual color dimorphism observed and that this dimorphism reflects different camouflage strategies, with highly mobile males showing a generalist coloration and more sedentary females showing a specialist coloration.

## Graphical abstract

The present study investigates color change, background choice, sexual color dimorphism, and movement rates in a praying mantis, *Stagmomantis limbata*. Nymphs changed color over development in response to rearing container color. Adults showed sexual color dimorphisms, which could potentially reflect different camouflage strategies.

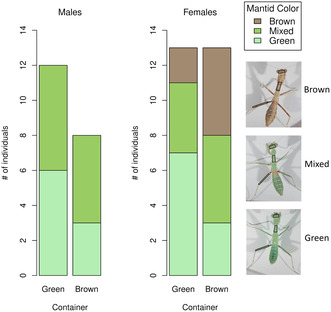

## 1 Introduction

Camouflage is important for animals that are hunted by visual predators, as well as for predators that must avoid detection to capture prey (Cuthill 2019; Pembury Smith and Ruxton 2020). Background matching is a common form of camouflage in which an animal's coloration matches its background color (Stevens and Merilaita 2009; Merilaita, Scott‐Samuel, and Cuthill 2017). For animals in homogeneous habitats, high levels of background matching can be achieved by matching the color of the habitat. But for mobile animals in habitats that vary in color over space or time, remaining matched against multiple potential backgrounds can be a challenge (Michalis et al. 2017; Hughes, Liggins, and Stevens 2019).

Animals have evolved several strategies for coping with spatial variation in background color (Caro and Koneru 2021). One solution is to change color via phenotypic plasticity, and this can reflect an underlying mechanism that is either physiological or morphological (Stevens 2016; Duarte, Flores, and Stevens 2017). Physiological color change, which involves pigment migration and redistribution, often takes place on a time scale of seconds to minutes and occurs in cephalopods, fish, and reptiles (Umbers et al. 2014; Duarte, Flores, and Stevens 2017). Alternatively, animals can evolve slower, morphological color change, involving synthesis, degradation, or modification of pigments (Stevens 2016), which typically occurs over hours to weeks and is common in crustaceans, insects, and vertebrates (Umbers et al. 2014; Kang, Kim, and Jang 2016). In some insects, morphological color change occurs only in association with molts (Edmunds and Brunner 1999; Noor, Parnell, and Grant 2008), perhaps because molting hormones also regulate switches from one color morph to another (Moriyama 2021). In other insects, morphological color change occurs independently of molts and can occur in adults (Umbers et al. 2014; Peralta‐Rincon, Escudero, and Edelaar 2017). A second strategy for crypsis in habitats with spatial color variation is background choice, wherein an animal chooses to rest on a substrate that matches its color (Stevens and Ruxton 2019). Background choice is expected to occur in animals with relatively slow color change or those with fixed body coloration (Stevens and Ruxton 2019). Background choice may, however, not be favored in species where other important benefits, such as securing key food resources or mating opportunities, outweigh the costs of reduced crypsis.

Cryptic body coloration can be broadly described as specialist or generalist, and the relative benefits of these two strategies depend on several factors (Merilaita, Tuomi, and Jormalainen 1999; Houston, Stevens, and Cuthill 2007; Nilsson and Ripa 2010; Hughes, Liggins, and Stevens 2019; Hughes et al. 2023). A specialist coloration matches one background color very well at the cost of being mismatched on others. A generalist coloration is one that is reasonably well matched on multiple background colors, either by adopting an intermediate blend of colors or by adopting blotches of different colors (Merilaita, Tuomi, and Jormalainen 1999; Hughes, Liggins, and Stevens 2019; Briolat et al. 2021).

An animal's movement patterns can influence the degree of habitat heterogeneity experienced and the effectiveness of different camouflage strategies (Bond 2007; Duarte, Stevens, and Flores 2016). Because morphological color change is often slow, usually occurring over days to weeks, it functions best for animals that do not move quickly through different background colors (Duarte, Flores, and Stevens 2017). The relative benefits of a generalist vs. specialist coloration also vary with mobility. Nilsson and Ripa (2010) compared the crypsis of prey with specialist vs. generalist coloration in a model with two habitat patches and found that greater movement rates between the patch types favored generalists. This is likely because when an animal's mobility is small relative to the scale of an environment's spatial color variation, the animal may be restricted primarily to a single background type, where a specialist coloration is most effective (Nilsson and Ripa 2010; Baling et al. 2020). However, when an animal's range of movement exceeds the scale of habitat color heterogeneity, the animal will be exposed to several different background colors, and a generalist coloration may become increasingly effective (Bond 2007; Nilsson and Ripa 2010; Briolat et al. 2021; Hughes et al. 2023). When optimal camouflage strategies are dependent on movement, this can manifest as sexual dimorphism in color within a species when differences in movement exist between the sexes (Taylor, Cook, and McGraw 2019; Cueva Del Castillo, González‐Zertuche, and Ramírez‐Delgado 2021).

In this study, we examine coloration, color change, camouflage strategies, and adult movement in a praying mantis, *Stagmomantis limbata* (Hahn), a species native to western North America and Central America (Maxwell 2014). *Stagmomantis limbata* consumes diverse insect prey and is itself subject to predation by spiders, birds, insectivorous mammals, and conspecifics (Maxwell and Frinchaboy 2014). Body color in *S. limbata* is variable, with individuals ranging continuously from shades of green to brown, and some individuals exhibiting multiple colors (Roberts 1937; Maxwell 2014). Color change has been informally described in nymphs (Roberts 1937). Adult females are flightless and sedentary, whereas adult males have longer, functional wings and fly in search of receptive females (Rau and Rau 1913; Maxwell and Frinchaboy 2014). Thus, this system is well suited for finding whether differences in mobility between the sexes might be associated with sexual differences in body coloration. We address the following questions: (1) Do nymphs change color during development, and if so, is this color change influenced by the background color on which they are reared? (2) Does color change only occur in association with molts or can it occur within an instar? (3) Do adults exhibit sexual color dimorphism? (4) Do adults exhibit background choice? and (5) Do adult males and females show differences in mobility in the field.

## 2 Materials and Methods

### 2.1 Color Change Experiment

In this experiment, we reared nymphs from hatchlings to adults in either a green or brown rearing container and photographed nymphs over their development to track their color. This experiment was used to (1) examine patterns of nymphal color change over development, (2) test if nymphs change color between vs. within instars, (3) test if the rearing environment color influences mantid color, and (4) test for adult sexual color dimorphism.

#### 2.1.1 Rearing

We collected mantid oothecae (*N* = 4) from gardens in a residential neighborhood in Davis, California (USA) (38.547942° N, 121.781823° W) and placed them in a growth chamber (24°C, photoperiod 14.5:9.5 L:D) on 23 February 2020. Oothecae were checked daily for emergence. A total of 448 mantids hatched during 18 March—2 April. Upon hatching, each nymph was randomly assigned to a rearing color treatment: Green or brown container. Forty‐five hatchlings (11–12 hatchlings from each ootheca) were reared individually (one hatchling per container) to track the color of individual nymphs starting in the first instar. All other hatchlings (*N* = 403) were reared in groups (*N* = 40 groups) with 2–15 hatchlings per container. Group containers contained hatchlings from the same ootheca and hatching day; this allowed us to keep track of which ootheca each nymph hatched from and to test for potential genetic or maternal effects on nymph color. Group rearing was used in order to begin the experiment with a large number of nymphs (since we were concerned about the possibility of high mortality in the early instars). The number of nymphs per group‐rearing container was reduced to three individuals at 19 days post‐hatching (during the second instar) and to one individual at 30 days post‐hatching (during the second and third instars). Retained nymphs were chosen randomly, and excess nymphs were released into their gardens of origin. All containers with hatchlings were maintained at 22°C from 18 March—1 April, and at 25°C thereafter, under natural lighting.

Rearing containers were clear, acrylic cubes (10 × 10 × 10 cm) with green or brown fabric (see Table A1 for RGB color values) glued to the walls, floor, and ceiling, and with a removable, mesh lid (Gary Plastic Packaging; Bronx, NY). The green and brown fabrics were the same material, but differed in hue and brightness, with the brown fabric having a darker shade than the green (Table A1). We drilled a 1 cm diameter hole in one side of each container where we attached a 200 mL vial for introducing prey. Mantids in the first three instars were fed small laboratory‐reared *Drosophila* spp. *ad libitum*. Fourth and fifth instar nymphs were fed one to two house cricket nymphs (*Acheta domestica*) per day and flightless *Drosophila hydei ad libitum*. Sixth and seventh instars and adults were fed two to four cricket nymphs per day. A small streak of honey was placed on the container lid to feed the *Drosophila* flies. To provide hydration, we brushed water on the mesh lids twice per day until 3 May and subsequently sprayed each lid with water once per day.

Mantids were sexed at the early fifth instar by examining wing pad formation and the abdominal terga. Mortality was observed during rearing. Fifty‐one mantids survived to the fourth instar (23 reared individually, 28 initially group‐reared), and twenty‐nine mantids survived to adulthood: 17 females and 12 males (12 reared individually, 17 initially group‐reared).

#### 2.1.2 Quantifying Mantid Color

Mantids were photographed in a standardized arena to track the color changes of individuals across development. For mantids reared individually, photography began in the first instar, 10 days post‐hatching. Because group‐reared nymphs in the same containers could not be distinguished from one another, we did not begin photography of group‐reared nymphs until the fourth instar, at which point nymphs had been reduced to one per container. Mantids were photographed at two time points in each instar, early and late, so that color change both within and across instars could be examined. The early photo was taken 1 day after each molt (to allow for sclerotization of the exoskeleton). The late photo was taken near the end of the instar, 8 days after each molt (two exceptions were made: The late photo was taken at 10 days for the first instar, because it was generally longer, and 7 days for the fourth instar, because it was shorter).

Mantids were photographed inside a 26 × 36 × 36 cm “white box” lined with white paper. Fiber–optic lights (Intralux 5000) were inserted through holes in the sides of the box. A camera (Canon Rebel T7i DSL) was positioned with an overhead view of the interior of the box. To photograph a mantid, we removed the mantid from its container, placed it on a clear plastic lid, and slid the lid onto a raised platform inside the white box (we did not perform any procedures to immobilize nymphs prior to photography). After the fourth instar, multiple photographs of each mantid were needed to capture all body parts in focus. We took photographs with constant lighting, focal distance (60 mm), aperture (f/5.6), ISO (400), and shutter speed (1/125 s). Custom white balance was set by photographing a white printer paper.

**FIGURE A1:**
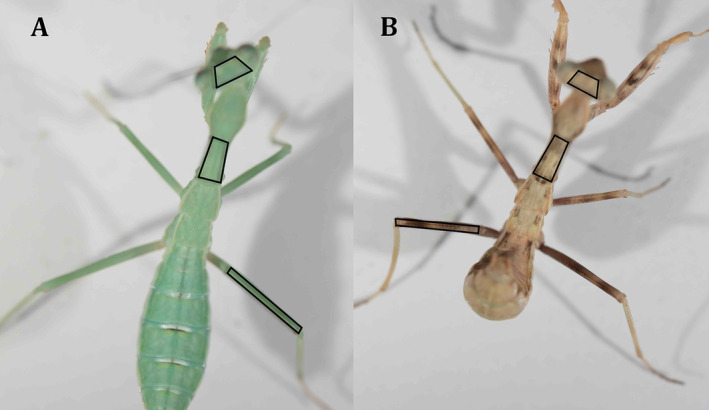
Three body regions (head, posterior half of pronotum, and metathoracic femur) for which RGB values were extracted for nymphs, shown in black outline. Panel (A): Green fourth instar female with relative redness of head = −0.075, pronotum = −0.071, femur = −0.079; average of these values = −0.075. Panel (B): Brown fourth instar female with relative redness of head = 0.183, pronotum = 0.184, femur = 0.315; average = 0.227.

Photographs were taken in RAW format, converted to 8 bit TIFF, and imported into imageJ for the extraction of the RGB color channels (Troscianko and Stevens 2015). Some mantids showed variation in color across body structures. To measure this variation, we measured the RGB values for three separate body regions—the head, the posterior half of the pronotum, and the metathoracic femur—by selecting the area of each body region (as shown in Figure A1) and calculating the average RGB for all pixels in this area. In adults, RGB values were measured for a fourth body region, the forewing.

Because we did not immobilize nymphs prior to photography, mantises often varied in their exact position relative to the light source in the photographic arena, which caused variation in the brightness of mantid illumination. This variation caused the measured RGB values (which measure hue and brightness) to have low repeatability (reported below). We were able to correct for this by converting these measured RGB values (*R*_meas_, *G*_meas_, and *B*_meas_) to relative RGB values (*R*_rel_, *G*_rel_, and *B*_rel_). Relative RGB values measure only hue and were highly repeatable (reported below). This conversion to relative RGB was performed separately on each triad of measured RGB values obtained for a mantid body region as follows:

First, we calculated the average light intensity across each measured RGB triad, $\bar{I}$:

$$\bar{I}=\frac{R_{\text{meas}}+G_{\text{meas}}+B_{\text{meas}}\;}{3}$$

Then, using $\bar{I}$, we calculated the relative values:

$$R_{\mathrm{rel}}=\frac{R_{\text{meas}}-\bar{I}}{\bar{I}}$$

$$G_{\mathrm{rel}}=\frac{G_{\text{meas}}-\bar{I}}{\bar{I}}$$

$$B_{\mathrm{rel}}=\frac{B_{\text{meas}}-\bar{I}}{\bar{I}}$$

The repeatability of measured RGB values and relative RGB values was calculated using a repeatability analysis using the R package *rptR* (Stoffel, Nakagawa, and Schielzeth 2017). We used data from pairs of photos (*N* = 158) of the same mantid taken successively on the same day. These pairs of photos included mantids at all developmental time points. For each pair of photos, we measured the RGB values for the head, pronotum, and femur and compared the values obtained from photo 1 with those obtained from photo 2. The repeatability analysis measures how similar the measurements are within each pair of photos. Repeatability values range from 0 to 1, with 0 indicating that the measurements are not repeatable whatsoever and 1 indicating that the measurements are identical to each other and maximally repeatable; we sought repeatability values > 0.90. The repeatability of the measured RGB values was unsatisfactory: Repeatability ± SE = 0.84 ± 0.03, 0.79 ± 0.04, and 0.71 ± 0.06 for *R*_meas_, *G*_meas_, and *B*_meas_, respectively. However, relative RGB values were highly repeatable (repeatability ± SE = 0.99 ± 0.003, 0.99 ± 0.003, 0.94 ± 0.01 for *R*_rel_, *G*_rel_, and *B*_rel_, respectively) and thus were used to quantify the mantid color in our study.

**FIGURE A2:**
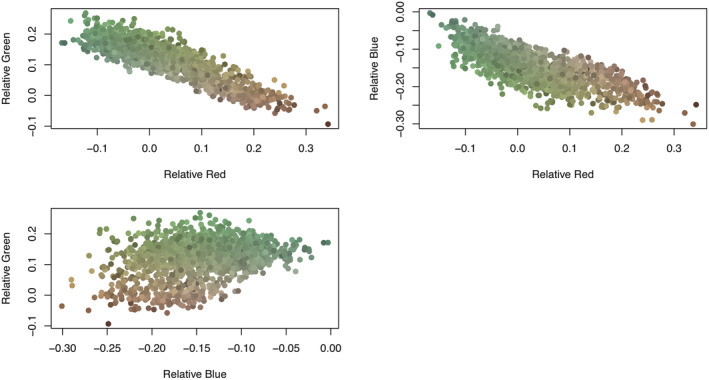
Correlations between the three relative RGB color channels. Data are RGB values extracted from all mantids for all developmental stages. Correlations: Relative redness and relative green (*r* = −0.86, *p* < 0.0001, *N* = 1494); relative redness and relative blue (*r* = −0.77, *p* < 0.0001, *N* = 1494); relative green and relative blue (*r* = 0.34, *p* < 0.0001, *N* = 1494).

*R*_rel_, *G*_rel_, and *B*_rel_ were highly correlated (Figure A2). We found that relative redness alone was a good univariate measure of color because brown mantid regions had high relative redness, and green regions had low relative redness (Figure A1); similarly, the brown fabric had high relative redness (*R*_rel_ = 0.281) and green fabric had low relative redness (*R*_rel_ = −0.409, Table A1). For some analyses, it was convenient to combine the relative redness values of the different body regions of a mantid into a single average relative redness value for each individual at that point in time. Average relative redness was calculated by taking the mean of the relative redness values from each of the body regions measured (for nymphs, the head, pronotum, and femur; for adults, the head, pronotum, femur, and forewing). We refer to this metric as “average relative redness.”

#### 2.1.3 Color Change Over Development

To examine whether color change is associated with molting, we compared the rate of color change within an instar versus between instars using a linear mixed‐effect model, where the absolute value of the rate of color change per day was the response variable, time interval (between vs. within an instar) was a fixed‐effect predictor, ootheca ID was a fixed effect, and mantid ID was a random effect to account for repeated measures. *p*‐values were obtained from the linear mixed‐effect model using the Satterthwaite approximation from the *lmerTest* package (Kuznetsova, Brockhoff, and Christensen 2017; Luke 2017) in R. The absolute value of the rate of color change within an instar was calculated as the absolute value of the difference between the average relative redness of a nymph early in an instar (day 1) and late in that instar (usually day 8, but see above for exceptions), divided by the number of days between the two measurements. The rate of color change between instars was calculated as the absolute value of the difference between the average relative redness of a nymph late in an instar (usually day 8) and early in the succeeding instar (day 1), divided by the number of days between the two measurements. We used the absolute value of color changes because we wanted to quantify the magnitude of color change occurring between vs. within instars, rather than the direction of this color change.

#### 2.1.4 Effects of Rearing Container Color on Mantid Color

To assess whether the mantid color was influenced by the rearing container color, we performed two separate analyses. For the first analysis, we used a linear mixed‐effect model to test whether the relative redness of the body regions was influenced by the rearing container color. Ootheca ID, sex, and initial rearing density (reared individually vs. in groups) were also included as fixed effects. We performed this test at two developmental stages: The late fourth instars (when our sample size was largest, *N* = 51) and adults (the end of the color change experiment, *N* = 29). Because the response variable was the relative redness of multiple body regions (head, pronotum, and femur; for adults, forewing was also included), we included “body region” as a fixed effect and mantid ID as a random effect to account for repeated measures. *p*‐values were obtained using the Satterthwaite approximation. In our first analysis, we chose to avoid taking color measurements of the most proximal portion of the femur because the mantid's body often cast a shadow over this area. However, we subsequently observed that the base of the femur often has brown pigmentation in otherwise green nymphs, so we conducted a second analysis that addressed this color variation. Two observers (LR and JR) blindly (i.e., without knowledge of treatment assignments) and independently categorized mantids as either uniform green, mixed (green with some brown pigmentation) or brown. Color categorizations by LR and JR were identical for 48 of 51 nymphs. Using a cumulative link model from the *MASS* package (Venables and Ripley 2002) in R, we analyzed if mantid color categorization was influenced by the fixed effects of rearing container color, ootheca ID, sex, or initial rearing density (reared individually vs. in groups). We performed this test twice, with LR color categorization or JR color categorization as the response variable, respectively.

#### 2.1.5 Adult Sexual Color Dimorphism

We examined color variation within and between adults. To measure the within‐individual variation, we calculated the standard deviation of relative redness for each adult's four measured body regions (head, pronotum, femur, and forewing). We used a linear model to find whether the standard deviation of relative redness within individuals was influenced by the mantid sex or ootheca ID. To test whether the between‐individual variation differed by sex, we used Levene's test for homogeneity of variance. We also ran Levene's test a second time, with ootheca ID as the predictor.

### 2.2 Background Color Choice in Adults

**FIGURE A3:**
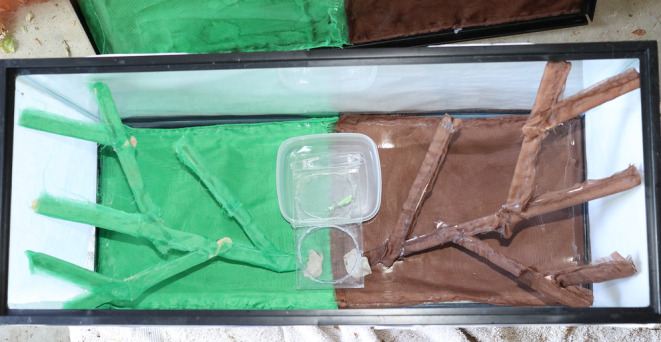
Arena for background choice assays.

We conducted an experiment to test whether adult mantids choose a background that matches them in color. All mantids reared to adults in the color change experiment were assayed (*N* = 29; 17 females, 12 males). We constructed two identical choice arenas, each placed in a shaded outdoor area 20 m apart. Each arena was a glass terrarium (38 cm × 76 cm, 38 cm high) divided into two halves. Each half was covered with either green or brown fabric on the floor and ceiling (the same fabric as in the rearing containers); the outside walls of the terrarium were covered with white paper. Each arena half contained a standardized branched substrate covered with green or brown fabric (Figure A3).

Assays were conducted during 1–30 June 2020, with one assay per day per arena. Each mantid was photographed 1 day before the assay to quantify its color. Mantids were introduced into the center of the arena at 10:00 h on a plastic, clear stage (8 cm × 8 cm). Of all mantids assayed, only one entered one side of the arena within the first 2 min, suggesting that choice was not made rapidly due to the possible stress associated with handling. Mantid location was recorded at 2‐h intervals until 16:00 h (i.e., 6‐h assay duration). To test whether the mantid location in the arena was influenced by the mantid body color, we ran a logistic regression with a binary response variable of mantid location at the green or brown side at the end of the assay (16:00 h), with the mantid average relative redness, arena ID, ootheca ID, rearing container color experienced, and sex included as fixed effects. In five assays, mantids remained at the release location for the entire duration (no choice made); these mantids were excluded from analysis. After each assay, the entire contents of each terrarium including all fabric were thoroughly cleaned with soap and water and allowed to fully dry overnight, to remove possible chemical cues. To check for the consistency of mantid choice, 16 randomly selected adults were assayed a second time, with at least 1 day between assays, and an asymptotic generalized Cochran–Mantel–Haenszel test was performed to find whether the mantid choice at 6 h in the second (repeat) assay was influenced by the original mantid choice at 6 h in the first assay.

### 2.3 Adult Mobility in the Field

We monitored adult mantids in the field to compare the mobility of males and females. From 7 September to 13 November 2020, we located and marked adult mantids in two large communal gardens (25 m × 110 m and 25 m × 320 m; *N* = 12 individuals) and five smaller gardens (*N* = 47 individuals) in a residential neighborhood in Davis, California (USA) (38.547942° N, 121.781823° W). All mantids found were marked with a unique combination of colored spots on various body locations (upper, mid or lower pronotum, and forewing) using permanent markers (Newell Brands, Sharpie). Mantids were marked by touching them gently with the markers and without otherwise handling them. For 6 of 7 days per week, mantids were checked once per day between 10:00 and 16:00 h from 7 to 28 September and twice per day from 29 September—13 November. For each check, one researcher (LR) visually searched for the mantid at its last location for a maximum of 3 min. The radius of the search area was 2 m. For each mantid found, the date, location on plant, and distance from its last observed location were recorded. If the mantid was not found after 3 min, we recorded that it was missing. If a mantid was not found for 3 consecutive days, we conducted checks 1 and 2 weeks later and then ceased searching the location.

We quantified mantid movement in two ways. First, we measured the net movement per day of adult mantids across daily censuses (net distance moved divided by the number of days between sightings). Because net movement distances were not normally distributed, we rank‐transformed the data and then used a linear mixed‐effects model to test if the mantid movement was influenced by sex, with the mantid ID as a random effect. Second, we indirectly measured longer‐range mantid movements by calculating “days until final observation,” the number of days between a mantid's first and last sighting. This was an indirect measure of longer‐range movements because the occurrence of a mantid's last sighting (such that we did not find it on all subsequent searches) is very likely to be the result of the mantid moving outside the census area (at least 2 m), although we cannot rule out mortality events or failure to resight a mantid that had not moved (Appendix A). We used a survivorship analysis from the *survival* package (Therneau and Grambsch 2000) in R, to find whether days until the final observation was influenced by sex. Four males were observed being cannibalized by females and were not included in the analysis.

Mean ± 1SE values are reported throughout the results. All statistical analyses were conducted with R version 4.3.2 (R Core Team 2021).

## 3 Results

### 3.1 Color Change Over Development

**FIGURE 1:**
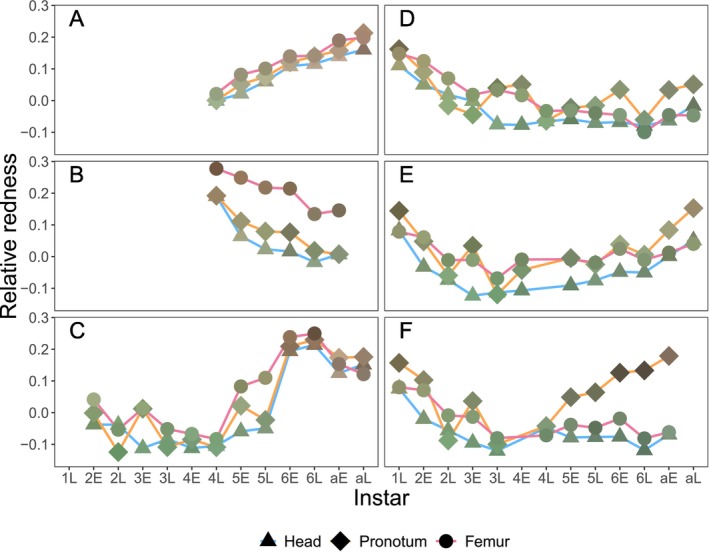
Examples of color changes over development for *S. limbata* in three females (A–C) and three males (D–F) (Nymphs in (A, B) were initially group‐reared, so photos of color began at the late fourth instar). *x*‐axis: Instar (first to sixth, “a” = adult) and time within instar (E = early, 1 day post‐molt; L = late, 8 days post‐molt). *y*‐axis: Relative redness of three body parts (head = triangle, pronotum = diamond, femur = circle), where higher relative redness indicates browner and lower relative redness indicates greener. The color of each symbol indicates the actual color of each body part. To view the color trajectory of each mantid in the experiment, see Figure A4. To view color trajectories separated by ootheca ID, see Figure A10.

**FIGURE A4:**
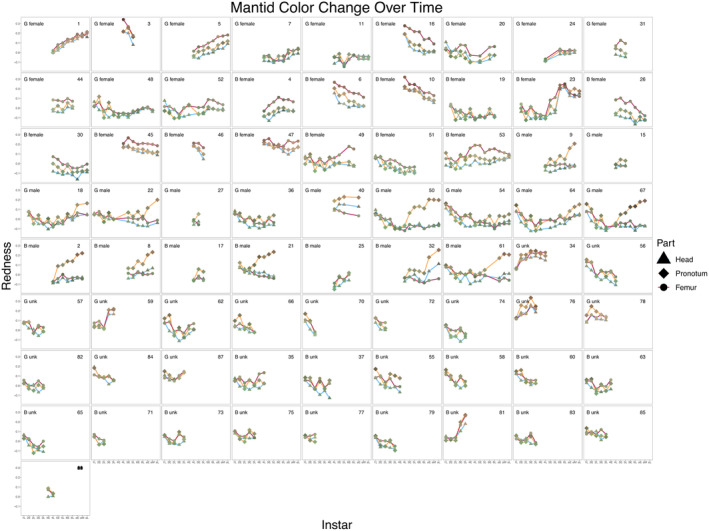
Color trajectories for all individual nymphs in across development. *x*‐axis: Development stage (instar number, with early (E) or late (L) within each instar). *y*‐axis: Relative redness of three body parts: The head (triangle), pronotum (diamond), and femur (circle). Mantid rearing container color (G = green, B = brown) and sex are given in the top left corner of each panel.

**FIGURE A5:**
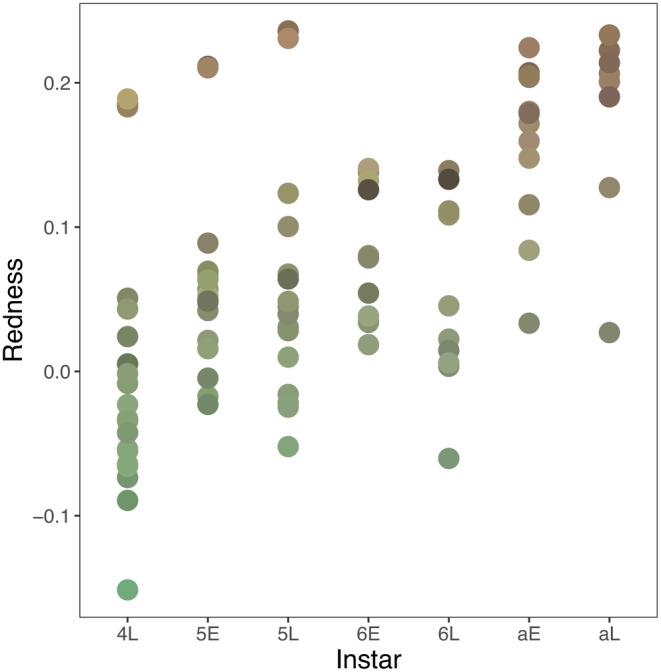
Males: Change in pronotum color across development (E = early within stage, L = late within stage, a = adult).

**FIGURE A6:**
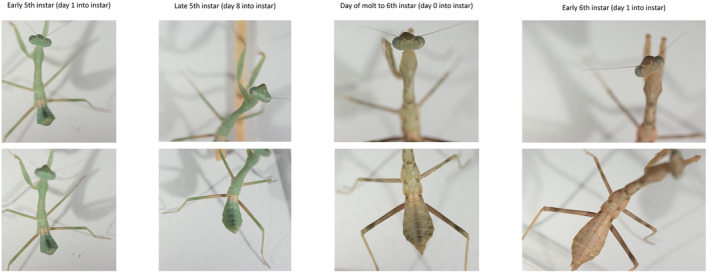
Example of rapid color change associated with the molt to the sixth instar in an individual nymph (at each time point, two photos of the mantid were taken to capture the full mantid's body in view). In the fifth instar, the brown pigmentation on the femur became more pronounced, but otherwise the nymph was green. Upon molting to sixth instar (day 0), the nymph is intermediate greenish‐brown in overall coloration. By day 1, the nymph is fully brown. This nymph is the same as shown in Figure 1C.

Mantids in the color change experiment, in both green and brown containers, exhibited substantial color changes over the course of development (Figure 1, Figures A4, A5, A6). Figure 1 shows a subset of color trajectories for three females (A–C) and three males (D–F); the individual color trajectories of all nymphs are shown in Figure A4. Some mantids became greener (Figure 1B,D), others became browner (Figure 1A,C), and in still other cases the trends in color change reversed (Figure 1E,F). Also, mantids showed variation in color across different body regions. Males often developed a brown pronotum while maintaining otherwise green bodies (Figure 1E,F, Figures A4, A5). Also, green nymphs of both sexes often developed brown pigmentation on their femurs (e.g., Figure 1B, Figure A4) and on the tergum of the first abdominal segment.

**FIGURE 2:**
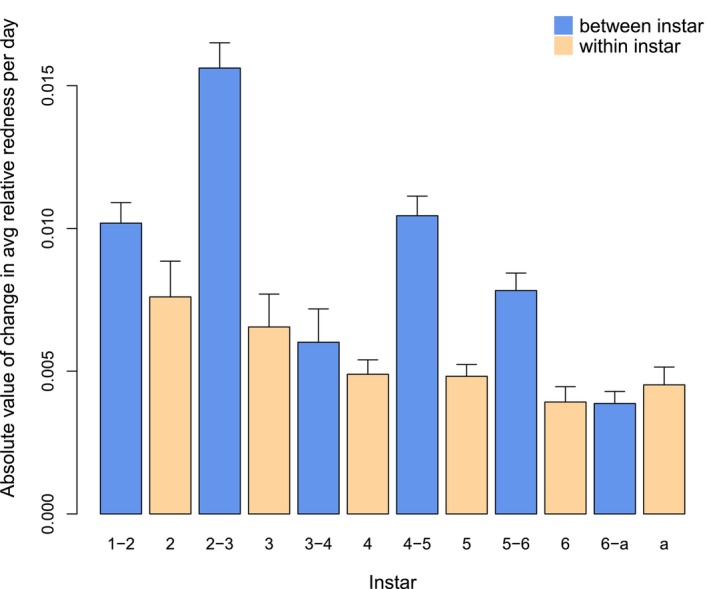
Rate of color change per day within (tan) and between (blue) instars. *x*‐axis: Stage (first to sixth instar, “a” = adult). *y*‐axis: Absolute value of the change in average relative redness per day (mean + SE).

The rate of color change was highly variable. Most individuals showed gradual change over the course of several instars (Figure 1A,B, Figure A4), but some showed abrupt changes. The most dramatic changes in color occurred when individuals jumped from predominantly green to brown over a single molt (Figure 1C, Figure A6). The rate of color change between instars (i.e., across molts) was significantly greater than the rate of color change within an instar (Figure 2, linear mixed‐effect model, coefficient for between instars = 0.0041 ± 0.0006, *F*_1,318_ = 51.9, *p* < 0.0001; see Table A2 for full model statistics). Ootheca ID had no effect on the rate of color change (*F*_3,71_ = 0.68, *p* = 0.57).

**FIGURE A7:**
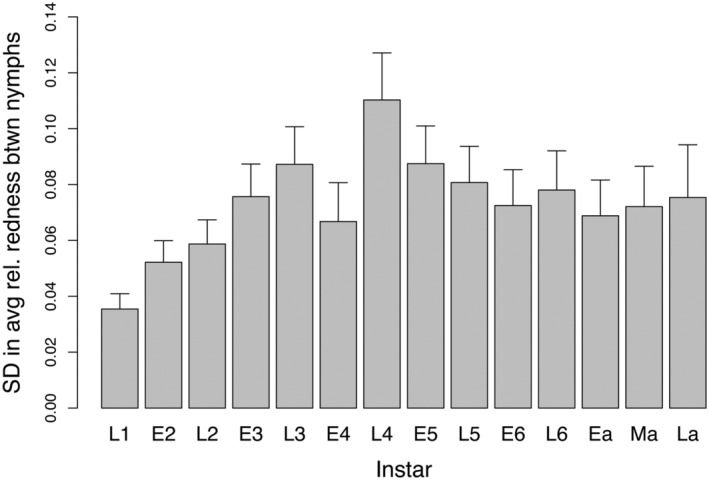
Between‐individual variation (measured as standard deviation) in average relative redness at different developmental stages. Instar number given; a = adult; E = early within stage; L = late within stage; M = middle within stage.

**FIGURE A8:**
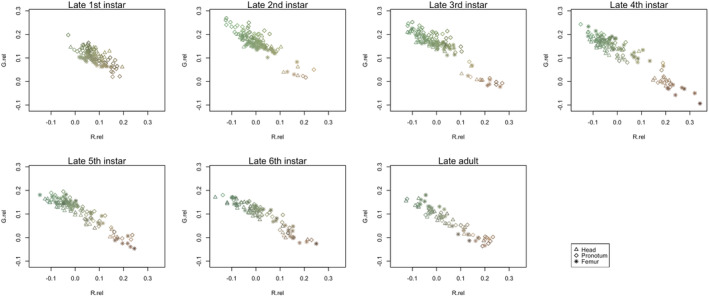
Plots showing variation in color of nymphs at different developmental stages. For each nymph, the relative redness and greenness of each of its three body regions are plotted: The head (triangle), pronotum (diamond), and femur (star). *x*‐axis plots relative redness and *y*‐axis plots relative greenness.

**FIGURE A9:**
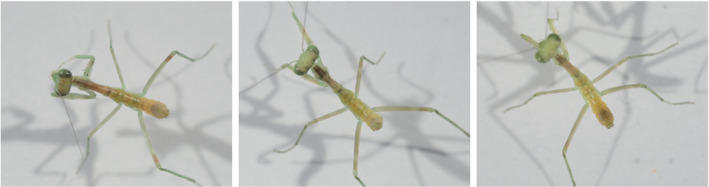
Three randomly selected hatchlings from the same ootheca, on the day they emerged from the ootheca.

We observed substantial changes in between‐individual variation in color over development (Levene's test, *F*_13,484_ = 2.418, *p* = 0.004, Figures A7, A8). Late first instars were more similar in average relative redness to each other than any other developmental stage; they had an intermediate, pale greenish‐brown coloration (Figures A8, A9). Also, the average relative redness of a late first instar was not correlated with its average relative redness at the late third instar (linear model, *F*_1,36_ = 1.2, *p* = 0.28; our sample size was too small to compare across larger time intervals). Thus, nymphs that were slightly browner on average in the first instar were not more likely to turn brown later.

### 3.2 Effects of Rearing Container Color on Mantid Color

**FIGURE A10:**
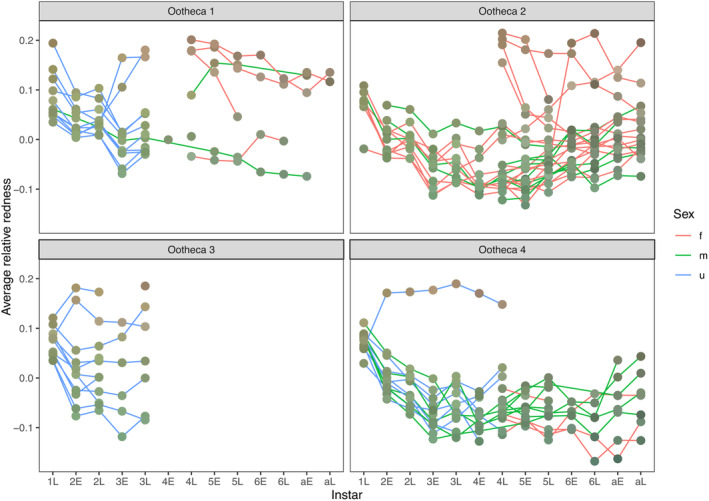
Mantid color change over development, separated by ootheca. Each panel shows the color trajectories for nymphs that emerged from that ootheca. Mantid sex is shown by line color (red = female, green = male, blue = unknown). Nymphs in ootheca 1 became significantly browner than nymphs in ootheca 2 or 4. Nymphs from ootheca 3 developed infection symptoms, and none survived to the fourth instar.

We examined the effect of rearing container color on mantid color using two separate analyses. In the first analysis, mantid relative redness of the head, pronotum, or femur (and in adults, also the forewing) was not influenced by the rearing container color (linear mixed‐effect model with Satterthwaite approximation; late fourth instars: *F*_1,44_ = 0.60, *p* = 0.44, Table A3; adults: *F*_1,23_ = 1.01, *p* = 0.33, Table A4). The source ootheca had a marginally nonsignificant influence on relative redness for late fourth instars (*F*_2,44_ = 3.06, *p* = 0.057) but a strong influence for adults (*F*_2,23_ = 7.84, *p* = 0.0025, Figure A10). Relative redness was also significantly influenced by the body region, with a higher relative redness in the femur and pronotum than in the head and forewing (late fourth instars: *F*_2,100_ = 29.1, *p* < 0.0001; adults: *F*_3,84_ = 12.3, *p* < 0.0001). There was no effect of mantid sex (late fourth instars: *F*_2,44_ = 2.33, *p* = 0.11; adults: *F*_1,23_ = 0.18, *p* = 0.67) or initial rearing density (group vs. individually reared; late fourth instars: *F*_1,44_ = 2.01, *p* = 0.16; adults: *F*_1,23_ = 0.02, *p* = 0.89) on relative redness.

**FIGURE 3:**
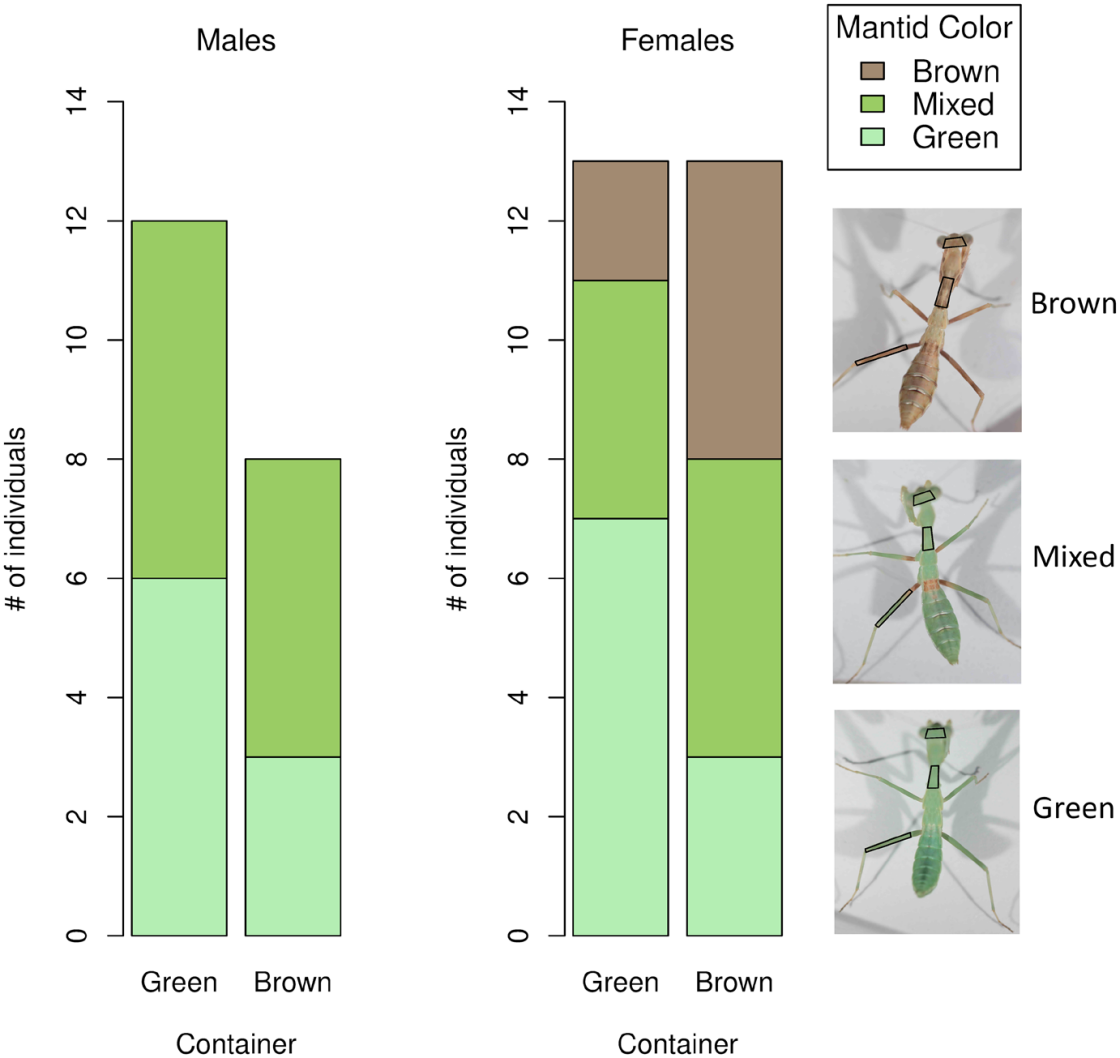
Test of the influence of rearing container color on mantid color. *x*‐axis: Rearing container color (green or brown) for late fourth instar males and females (five mantids that were not yet sexable are not shown). *y*‐axis: The number of mantids categorized as green, mixed, or brown (data from categorization by LR). On the right: Examples of green, mixed, and brown fourth instars, with body regions where color was measured outlined (head, pronotum, and metathoracic femur).

In the second analysis, blind assignment of late fourth instars to the color categories of green, mixed, or brown by two observers revealed a significant effect of rearing container color (Figure 3; cumulative link model, *N* = 51, *t* = 2.03 for LR and *t* = 2.13 for JR, *p* = 0.042 and 0.033, respectively). Ootheca ID also significantly influenced mantid color categorization, but the initial rearing density and sex had no effect (Table A5). Females appeared to be more responsive to rearing container color than males (Figure 3), but our limited sample size precludes formal analysis. Thus, although the first analysis showed no effect of rearing container color on the mantid color, the second analysis did; we address these conflicting results in Discussion.

### 3.3 Adult Sexual Color Dimorphism

#### 3.3.1 Within‐Individual Variation in Body Color

**FIGURE 4:**
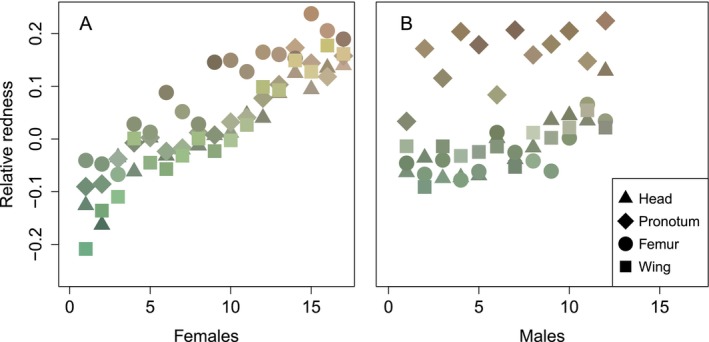
Sexual color dimorphism of adult females (A) and males (B). Each individual mantid is plotted at a single location on the *x*‐axis, ranked from greenest (*x* = 1) to brownest (*x* = 17 for females; *x* = 12 for males). *y*‐axis: Relative redness of the four measured body parts (head = triangle, pronotum = diamond, femur = circle, wing = square).

**FIGURE 5:**
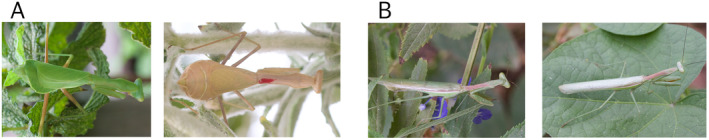
Examples of two adult female (A) and male (B) *S. limbata*. Individual females are relatively homogeneous in color but vary continuously between individuals from green to brown (the red marking on the pronotum of one female is from a permanent pen). In contrast, individual males are heterogeneous in color (with a green head, legs, and wings, but a brown pronotum), but between individuals males share a similar coloration.

Adult mantids raised in the color change experiment exhibited sexual color dimorphism (Figures 4, 5). Females showed significantly lower within‐individual variation in color than males (standard deviation in relative redness of each body region for females = 0.045 ± 0.005, males = 0.091 ± 0.009, linear model, *N*_females_ = 17, *N*_males_ = 12, *t* = 4.57, *p* = 0.0001, Figure 4); ootheca ID had no effect (*t* = −0.168, *p* = 0.87). For females, the four measured body regions were similar in color (being green, brown, or any color in between), whereas for males, the head, femur, and wings were green, but the pronotum was generally brown (Figures 4, 5).

#### 3.3.2 Between‐Individual Variation in Body Color

Ootheca ID had no effect on between‐individual variation in body color (Levene's test, *F*_2,26_ = 0.11, *p* = 0.90). In contrast, sex had a strong influence on between‐individual variation: Females had significantly higher between‐individual variation in average relative redness than did males (standard deviation of average relative redness between females = 0.090, between males = 0.037, Levene's test, *F*_1,27_ = 8.66, *p* < 0.007, Figure 4). Females varied continuously in average color from green (Figure 4A at *x* = 1) to brown (Figure 4A at *x* = 17). In contrast, males varied less in average color, with most converging on a common phenotype of green body parts and a brown pronotum (Figures 4, 5).

### 3.4 Background Choice in Adults

Adult mantids did not choose backgrounds (green vs. brown side of arena) that matched them in color. Choice of background at the end of the 6‐h choice assay was not significantly influenced by mantid color, measured as average relative redness, or any of the covariates (logistic regression, *N* = 24; average relative redness: *z* = 0.008, *p* = 0.99; rearing background color: *z* = −0.84, *p* = 0.4; sex: *z* = −0.02, *p* = 0.98; ootheca ID: *z* = 0.546, *p* = 0.59, or arena ID: *z* = 0.13, *p* = 0.89). There was also no overall mantid preference for one side of the test arena over the other at 6 h (binomial test, 11 of 24 adults on green side, *p* = 0.84). For the subset of 16 adults assayed twice, the color choice made in the second assay at 6 h was independent of the color choice made in the first assay at 6 h (asymptotic generalized Cochran–Mantel–Haenszel test, *χ* ^2^ = 2.5, df = 4, *p* = 0.64).

### 3.5 Adult Mobility in the Field

**FIGURE 6:**
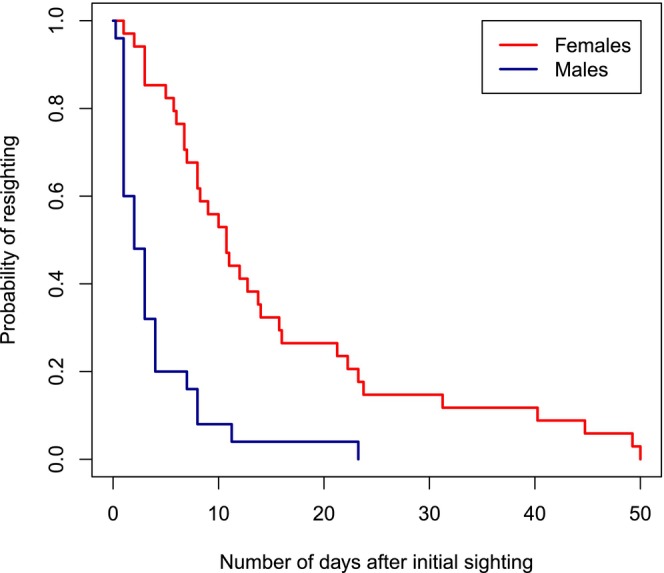
Probability of resighting adult male and female *S. limbata* in the field over successive days.

Adult males moved nearly twice as far per day (mean ± SE: 61.1 ± 19.9 cm/day, *N* = 41 total observations across 14 males) as did adult females (33.7 ± 3.0 cm/day, *N* = 318 total observations across 32 females; linear mixed‐effects model, *χ* ^2^ = 4.4, *p* = 0.036, Table A6). The maximum distance moved between sightings was 7.92 m for males and 5.96 m for females. Furthermore, the probability of resighting adult males across censuses was significantly lower than that for females (survivorship analysis, *N*_females_ = 34, *N*_males_ = 25, *χ* ^2^ = 24.9, df = 1, *p* < 0.0001; Figure 6). On average, females were observed for 15.3 ± 2.3 days before the final observation, whereas males were observed for just 3.8 ± 1.0 days.

## 4 Discussion

Our study quantifies the rate and patterns of color change over the course of development in the mantid *Stagmomantis limbata*. Nymphs are capable of changing color from green to brown, and vice versa, over development. Despite conflicting results from the two analyses testing if nymphs change color in response to treatment, we suggest that rearing container color influenced mantid color (and we discuss this below). Adults exhibited sexual color dimorphism. Females were relatively uniform in color, ranging continuously from green to brown, demonstrating high between‐individual variation but low within‐individual variation in color. In contrast, males converged on a common phenotype of green head, femur, and wings, but a brown pronotum, demonstrating low between‐individual variation but high within‐individual variation in color. Adult males showed higher rates of daily movement and had a lower probability of being sighted across censuses than did adult females. Adults did not show evidence of choosing a background that matched them in color.

Animal color patterns can serve a variety of functions in different contexts, including camouflage, interspecific and intraspecific signaling, mate choice and sexual selection, thermoregulation, UV protection, and immune defense (Van Der Veen 2005; Caro, Sherratt, and Stevens 2016; Stuart‐Fox et al. 2021; Postema, Lippey, and Armstrong‐Ingram 2023). We focus on how the color patterns observed for these mantids might function in the specific context of camouflage and propose some testable hypotheses.

### 4.1 Body Color Plasticity

Consistent with the observations of Roberts (1937), *S. limbata* showed color change over development. Since color changes occurred on the scale of hours to weeks, we suggest that *S. limbata* employs morphological color change, involving the synthesis, degradation, or modification of pigments in the epidermis (Umbers et al. 2014; Figon and Casas 2018). Color change over development has also been informally described in many mantids (Rau and Rau 1913; Roberts 1937; James 1944; Ramsay 1990; Iwasaki 1992; Edmunds and Brunner 1999; Battiston and Fontana 2010; Maxwell and Frinchaboy 2014; Rodrigues et al. 2017; Burke and Holwell 2023). Ommochromes, pteridines, and tetrapyrroles have been found in the epidermis of mantids (Futahashi and Osanai‐Futahashi 2021), and some authors have suggested that the green color of mantids is caused by a tetrapyrrole pigment, biliverdin IX alpha, alone or in combination with a yellow carotenoid (Ramsay 1990; Shamim et al. 2014; Futahashi and Osanai‐Futahashi 2021).

### 4.2 Effect of Rearing Container Color on Mantid Color

**FIGURE A11:**
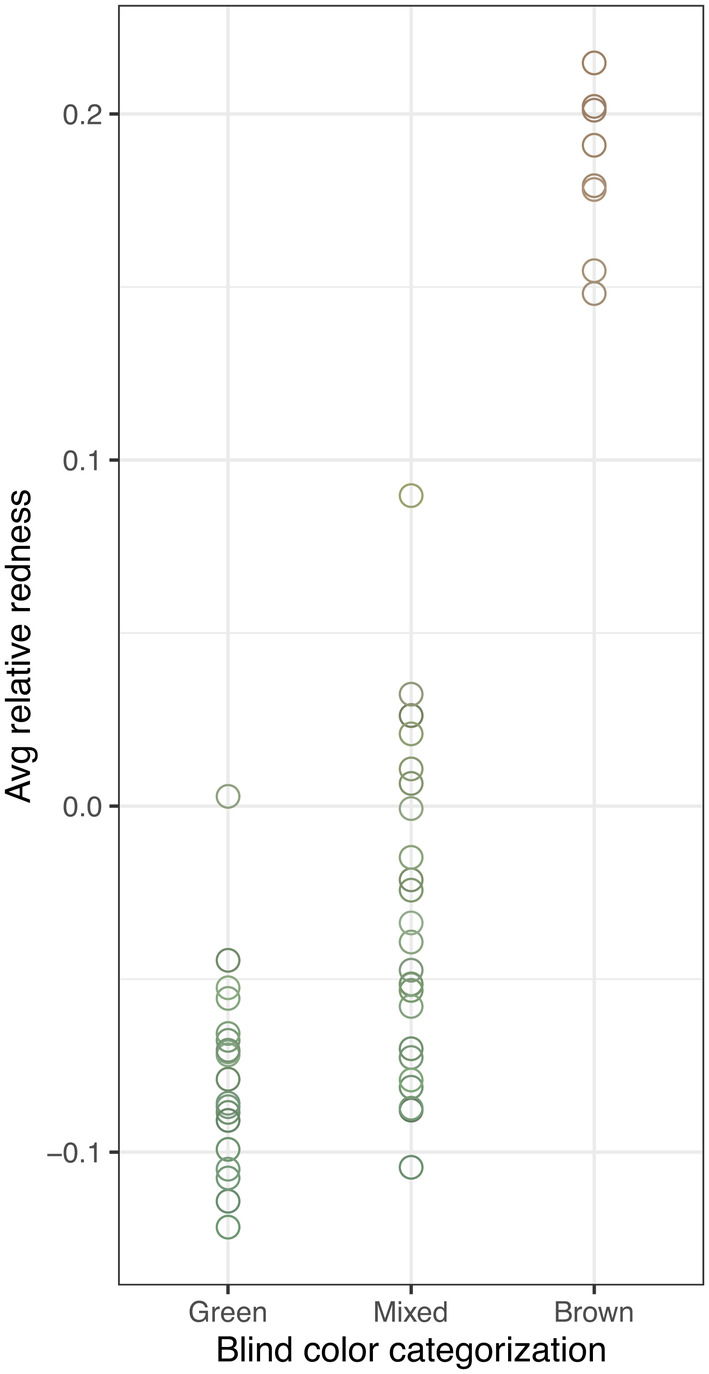
Color categorization (conducted without knowledge of the rearing container color treatment) of late fourth instar mantids vs. their average relative redness. About half of the mixed nymphs (green with some brown pigments) had average relative redness equivalent to the values for uniform green nymphs. Thus, average relative redness was not good at differentiating between nymphs that were green vs. mixed.

The two analyses examining the effect of rearing container color on mantid color yielded conflicting results. These differing results were likely generated by the alternative ways of describing body coloration. In both rearing container treatments, most nymphs were predominantly green, but 56% of these green nymphs contained brown coloration on the most proximal parts of their mid‐ and hind femora and on their first abdominal tergum (Figure 3). The first test did not measure the color of these specific body regions (Figure 3), so it lacked the information to effectively distinguish between green and mixed nymphs (Figure A11). In the second test, our visual assessment of mantid color included all regions of the mantid body, including the regions differentiating green versus mixed individuals. Thus, the second test had a greater ability to resolve rearing container color effects. We therefore conclude that the color of the rearing environment appears to influence mantid color, but the effect was noisy. We acknowledge that the darker shade of the brown fabric (Table A1) may have filtered more ambient light, and thus, the treatment effect might include both differences in rearing color and light intensity.

Aside from a recent study by Burke and Holwell (2023), few other studies have investigated whether nymphs change color in response to environmental factors such as background color (but see James 1944; Ergene 1953; Edmunds and Brunner 1999; Battiston and Fontana 2010). Our results are similar to those of Burke and Holwell (2023), which examined color change in the praying mantis *Miomantis caffra* and found a strong effect of rearing container color on mantid color, with most nymphs in green containers showing a green phenotype and nymphs in brown containers showing mostly a mixed or brown phenotype. Remarkably, the mixed phenotype of *M. caffra* matches the mixed phenotype of *S. limbata*: Predominantly green body coloration but with brown on the mid‐ and hind femora and on the first abdominal tergum.

Given that both *S. limbata* and *M. caffra* are capable of color change over development, it is curious that they develop predominantly a mixed phenotype against a brown rearing background. One possible explanation is that a mixed phenotype may be preferred over brown due to life‐history tradeoffs between camouflage and other functions of color (Van Der Veen 2005; Caro, Sherratt, and Stevens 2016; Stuart‐Fox et al. 2021; Postema, Lippey, and Armstrong‐Ingram 2023). Alternatively, Burke and Holwell (2023) suggest that a mixed phenotype might enhance crypsis against brown backgrounds via disruptive coloration, where a color pattern with contrasting colors functions to create false edges to disguise the body outline (Cuthill 2019).

The color change observed in *S. limbata* in response to rearing container color, although imperfect, lends support to the hypothesis that color change is an adaptation for improving crypsis. Color change in this mantid could be adaptive because nymphs may face environments that vary both spatially and temporally. When spatial variation in environment color occurs, with nymphs in different locations finding themselves in differently colored microhabitats, morphological color change could be quite beneficial. Color change in *S. limbata* could also be adaptive for coping with the temporal variation in background color, driven by changing seasons, as suggested for *Mantis religiosa* (Battiston and Fontana 2010). *Stagmomantis limbata* inhabits a Mediterranean climate, characterized by cool, wet winters, followed by hot, dry summers, and overall seasonal transitions in habitat color may occur, with the predominantly green vegetation in the spring changing to more brown substrates in the summer and fall.

Despite using only four oothecae in the present study, nymphal color was influenced by natal ootheca, suggesting that genetic or maternal effects may play a role in mantid color. If future research confirms a genetic component, then color phenotypes may be acted upon by natural selection, leading to local or global adaptation.

One relevant question is whether *S. limbata* can discriminate between different colors. Although some authors have suggested that mantids are monochromats (Fabricant and Herberstein 2015), relatively little is known about the spectral sensitivities of mantids (but see Sontag 1971; Rossel 1979). A definitive answer to this question requires additional characterization of mantid opsin genes.

### 4.3 Background Choice in Adults

Adult *S. limbata* showed no background choice, although we cannot discount the possibility that the artificial arena did not provide the necessary cues. Background choice may not occur for several reasons. First, it is unclear to what extent *S. limbata* can discriminate between different colors, and if it is a monochromat, this would constrain its ability to do background choice. However, supposing that *S. limbata* can distinguish between colors, background choice may not be adaptive for other reasons. For adult females, preference for a background that will improve crypsis could be detrimental if this leads females to reject areas with brightly colored flowers, which have high food availability. Also, because females are relatively sedentary, they may already possess an effective primary pathway to achieving camouflage, via color change. In males, background choice may be of limited benefit because they may have limited options for background selection when approaching and mating with females.

### 4.4 Adult Mobility in the Field

Both approaches used to estimate the mobility of adults indicated greater mobility for males in the field. Males moved greater distances across daily censuses and had a lower probability of being resighted across daily censuses, suggesting that they engage in long‐range movements more than females (see Appendix A for discussion of this metric). Mobility of adult mantids in the field has also been studied in *Tenodera sinensis*: Two studies of *T. sinensis* found that males move more than females (Bartley 1982; Christensen and Brown 2018), although one study found no significant difference (Eisenberg et al. 1992). The greater mobility in *S. limbata* males is consistent with the differences in the physiology of adults and their mating system. Adult females have reduced wings and are flightless, whereas males have longer wings and fly in nature (Maxwell and Frinchaboy 2014). Also, the mating system of *S. limbata* involves sedentary females releasing sex pheromones and males moving to find females, generally through a combination of longer distance flight, followed by shorter distance approach via crawling (Maxwell 1999; Maxwell, Barry, and Johns 2010; Maxwell, Gallego, and Barry 2010).

### 4.5 Sexual Color Dimorphism

Adult *S. limbata* exhibited sexual color dimorphism. Adult females varied dramatically between individuals in color but were relatively uniform in color within their bodies. In contrast, males were very similar in color but showed variation in color within their bodies, with a green body and a brown pronotum.

Sexual color dimorphisms can be driven by sexual selection or ecological selection, such as when differences in reproductive energetic needs or social roles of males and females lead to different optimal trait values, and the evolution of “dimorphic niches” in males and females (Slatkin 1984). While the observed sexual color dimorphism in *S. limbata* could be the result of several possible factors, we propose a hypothesis that the sex‐specific coloration may reflect differences in camouflage strategy. The convergence of males on a common coloration of a green body with a brown pronotum may indicate a generalist camouflage strategy, while the homogeneous body color of females, with different females exhibiting different colors, may reflect a specialist strategy. Indirect support for this hypothesis comes from the link between mobility and camouflage. Nilsson and Ripa (2010) showed with a model that high dispersal rates favor generalist coloration, while low dispersal rates favor specialist coloration. Thus, the higher mobility of adult males is consistent with the prediction that a generalist coloration could be favored, while the more sedentary behavior of adult females is consistent with a specialist coloration being favored.

To evaluate this hypothesis, further work is needed to investigate whether the male phenotype provides greater crypsis against a variety of backgrounds while the female phenotype is better matched to a single background. This could be tested via artificial simulation, by placing males and females on different background types for discovery by visual searchers, and comparing the rates of detection (e.g., Nokelainen et al. 2019; De Alcantara Viana et al. 2023; Hughes et al. 2023). Additional work is also needed to investigate other potential roles for sexual color dimorphism, such as differential habitat use, thermoregulation, or sexual selection.

Differences in mobility within the sexes appear to drive different camouflage strategies in other systems. In two grasshopper species, low‐mobility females employ a specialist background‐matching strategy, whereas high‐mobility males have greater disruptive coloration, which can improve crypsis on many differently colored backgrounds (Schaefer and Stobbe 2006; Ramírez‐Delgado and Cueva Del Castillo 2020; Cueva Del Castillo, González‐Zertuche, and Ramírez‐Delgado 2021). In three species of jumping spiders, males move greater distances than females for mate‐searching (Taylor, Cook, and McGraw 2019). In these species, females show a cryptic coloration, while male coloration and behavior appear to mimic stinging wasps, a strategy that can provide some protection from predators irrespective of the background type (Taylor, Cook, and McGraw 2019). In orchid mantids, low‐mobility adult females are white or yellow as part of an aggressive, flower mimicry strategy wherein females remain largely immobile to be undetected by pollinator prey or potential predators, whereas males have a more brown, cryptic coloration, likely due to the need for movement to find mates (Svenson et al. 2016). In a shrimp with two morphs—a transparent morph (exhibiting a generalist camouflage strategy) and an opaque/homogeneous morph (exhibiting a specialist strategy)—males were found to move greater distances than females and usually expressed the transparent, generalist morph type (Duarte and Flores 2017; Duarte, Stevens, and Flores 2016). These studies suggest that in multiple systems, greater mobility in males appears to favor the evolution of alternative camouflage strategies in the sexes, with males employing generalist coloration, disruptive coloration, or mimicry—strategies that likely aid crypsis against multiple background types.

Despite this research progress, predictions about the influence of movement on camouflage strategies remain to be systematically tested (Hughes, Liggins, and Stevens 2019; Caro and Koneru 2021). We suggest that a useful path to investigating the effect of mobility on optimal camouflage is to examine species in which mobility varies between the sexes. An advantage of this approach over comparative interspecific studies is that the sexes generally share more traits and environments, so factors contributing to differences in camouflage may be more likely to be identified. Differences in mobility in the sexes are common across many taxa, and thus many species can be candidates for such examinations.

## Author Contributions

**Leah Y. Rosenheim:** conceptualization (equal), formal analysis (equal), investigation (lead), methodology (equal), writing – original draft (lead), writing – review and editing (equal). **Jay A. Rosenheim:** conceptualization (equal), formal analysis (equal), investigation (equal), methodology (equal), resources (lead), writing – review and editing (equal). **Michael R. Maxwell:** conceptualization (equal), methodology (equal), writing – review and editing (equal).

## Conflicts of Interest

The authors declare no conflicts of interest.

## Supporting information


Data S1–S15.

## Data Availability

All data and R code are currently supplied as Data S1.

## Appendix A Days Until Final Observation: An Indirect Measure of Movement

We classified a mantid as “disappeared” after failure to find it on three consecutive days and on searches conducted 1 and 2 weeks later. In these cases, we recorded the first date at which we failed to resight the mantid as the date that it “disappeared.” There are three potential reasons for this “disappearance”: (i) movement—the mantid has moved from the area being searched, (ii) searching error—the mantid has not moved from the area being searched, but we failed to detect it, or (iii) a mortality event. Because disappearance can be caused by all three factors, greater male disappearance might not necessarily be a result of higher mobility. Below, we consider whether male mortality or investigator searching error could drive the trend of greater male disappearance.

Searching error did occur occasionally. For 21 of the 26 adult males, we found them every day, at every consecutive check, until they disappeared permanently. Of the other 5 males, we lost track of them for one or more days prior to their final disappearance 10 times. In 6 of these 10 times, we found the male within 2 m of where it was last seen. In 4 of the 10 times, we found the male over 2 m away: 2.3, 2.4, 5.6, and 7.6 m from the previously occupied location. This suggests a very low rate of searching error when males moved < 2 m. Thus, it is unlikely that searching error is the driver of greater rates of male disappearance.

Mortality in adult males and females was observed several times in the field. However, we do not think mortality is a likely driver of the male disappearance rates. In the field, 50% of males disappeared within 3 days of being found. Of the 26 males we observed in the field, 10 were seen for 1 day and disappeared on the next day. If such high rates of disappearance are due to mortality, then there would be a complete lack of adult males in the field within 1–2 weeks. However, contrary to this, we found relatively stable numbers of males in the field for several weeks, with male densities declining only later in the season, likely from cannibalism or old age. Thus, it seems highly unlikely that mortality alone is driving the extremely high rate of male disappearance.

**TABLE A1:** Measured RGB and relative RGB color values for the green and brown fabric used in the color change experiment and adult background choice assay.

Fabric color	Measured R	Measured G	Measured B	Avg measured RGB	Relative R	Relative G	Relative B
Green	83.9	200.8	141.1	141.9	−0.4087	0.4147	−0.0060
Brown	142.4	103.3	87.8	111.2	0.2813	−0.0709	−0.2105

*Note:* RGB values for the fabrics were measured from a photo of the background choice terrarium (Figure A3), with the two fabrics adjacent to one another, allowing for a side‐by‐side comparison. The photo was taken outdoors under natural lighting, and a custom white balance was set using white paper. The green fabric has a higher average measured RGB value (141.9) than the brown fabric (111.2), meaning that it is lighter in shade than the brown fabric. It is possible that the darker, brown fabric could block out more ambient light than the green fabric, which could lead to slight differences in the light intensity within the containers in the green vs. brown containers.

**TABLE A2:** Linear mixed‐effect model output for testing whether the rate of color change is influenced by fixed effects of time interval type (between vs. within an instar) or ootheca, with mantid ID as a random effect.

Variable	Sum of squares	Mean square	Numerator df	Denominator df	*F*	*p*
Time interval type (between instar or within)	0.00159	0.00159	1	318.3	51.9	4.229e−12
Ootheca	0.0000624	0.0000208	3	71.3	0.678	0.568

**TABLE A3:** Linear mixed‐effect model output for testing whether late fourth instar relative redness of nymphal body regions is influenced by treatment (rearing container color), ootheca, sex, body region, or initial rearing density, with mantid ID as a random effect.

Variable	Sum of squares	Mean square	Numerator df	Denominator df	*F*	*p*
Treatment	0.000813	0.000813	1	44	0.601	0.442
Ootheca	0.00827	0.00413	2	44	3.06	0.0572
Sex	0.00630	0.00315	2	44	2.33	0.109
Body region (head, pronotum, or femur)	0.0788	0.0394	2	100	29.1	1.07E−10
Initial rearing density	0.00272	0.00272	1	44	2.01	0.164

**TABLE A4:** Linear mixed‐effect model output for testing whether adult relative redness is influenced by treatment (rearing container color), ootheca, sex, body region, or initial rearing density, with mantid ID as a random effect.

Variable	Sum ofsquares	Mean square	Numerator df	Denominator df	*F*	*p*
Treatment	0.00373	0.00373	1	23	1.01	0.327
Ootheca	0.0583	0.0291	2	23	7.84	0.00253
Sex	0.000681	0.000681	1	23	0.183	0.672
Body region (head, pronotum, femur, or wing)	0.137	0.0458	3	84	12.3	9.27E−07
Initial rearing density	0.000073	0.000073	1	23	0.0196	0.890

**TABLE A5:** Statistical output for cumulative link model testing if color categorization by LR and JR of late fourth instars (as green, mixed, or brown) is influenced by treatment (rearing container color), sex, ootheca, or initial rearing density.

Color categorization by JR			Color categorization by LR		
Variable	*t*	*p*	Variable	*t*	*p*
Treatment	2.13	0.0329	Treatment	2.03	0.0424
Ooth ID 2	−1.90	0.0578	Ooth ID 2	−2.25	0.0247
Ooth ID 4	−2.04	0.0413	Ooth ID 4	−2.43	0.015
Sex Male	−0.637	0.524	Sex Male	−1.06	0.288
Sex Unknown	−0.401	0.688	Sex Unknown	0.156	0.876
Rearing density	−1.43	0.153	Rearing density	−1.65	0.0990

**TABLE A6:** Results for testing whether adult net movement per day (rank‐transformed) is influenced by the fixed effect of mantid sex, with mantid ID as a random effect.

Test type	npar	AIC	BIC	LogLik	Deviance	Chisq	df	*p*
lmer with only random effect	3	4334.4	4346.1	−2164.2	4328.4			
lmer with random and fixed effect	4	4332	4347.6	−2162	4324	4.420	1	0.0355

*Note:* Because this test only had one fixed effect (mantid sex) and one random effect (mantid ID), *p*‐values were obtained using the following method: We performed the lmer test with and without the fixed effect and then compared these two tests with ANOVA.
